# Hyperresponsiveness to social rewards in children and adolescents with attention-deficit/hyperactivity disorder (ADHD)

**DOI:** 10.1186/1744-9081-5-20

**Published:** 2009-05-08

**Authors:** Gregor Kohls, Beate Herpertz-Dahlmann, Kerstin Konrad

**Affiliations:** 1 Child Neuropsychology Section, Department of Child and Adolescent Psychiatry and Psychotherapy, RWTH Aachen University, Neuenhofer Weg 21, D – 52074 Aachen, Germany; 2 Center for Autism Research, Children's Hospital of Philadelphia, 3535 Market Street, 8th floor, Suite 860, Philadelphia, PA 19104, USA; 3 Department of Child and Adolescent Psychiatry and Psychotherapy, RWTH Aachen University, Neuenhofer Weg 21, D – 52074 Aachen, Germany

## Abstract

**Background:**

Current research suggests that attention-deficit/hyperactivity disorder (ADHD) is associated with larger behavioral sensitivity to reinforcement contingencies. However, most studies have focused thus far on the enhancing effects of tangible rewards such as money, neglecting that social-emotional stimuli may also impact task performance in ADHD patients.

**Methods:**

To determine whether non-social (monetary) and social (positive facial expressions) rewards differentially improve response inhibition accuracy in children and adolescents with ADHD, we applied an incentive go/no-go task with reward contingencies for successful inhibition and compared ADHD subjects with typically developing individuals.

**Results:**

Both social and monetary contingencies improved inhibition accuracy in all participants. However, individuals with ADHD displayed a particularly higher profit from social reward than healthy controls, suggesting that cognitive control in ADHD patients can be specifically improved by social reinforcement. By contrast, self-rated motivation associated with task performance was significantly lower in ADHD patients.

**Conclusion:**

Our findings provide evidence for hyperresponsiveness to social rewards in ADHD patients, which is accompanied by limited self-awareness. These data suggest that social reward procedures may be particularly useful in behavioral interventions in children with ADHD.

## Background

Recent models suggest that the core symptoms of attention-deficit/hyperactivity disorder (ADHD), namely inattention, hyperactivity and impulsivity, could result from dysfunction of two neurodevelopmental pathways: the executive and motivational pathways [1]. While the deficient executive pathway is assumed to involve an insufficient regulation of thought and action primarily characterized by a core deficit in inhibitory control, the dysfunctional motivational pathway is hypothesized to link behavioral symptoms, task engagement, and a biologically embedded alteration in reinforcement mechanisms. A number of studies investigated dysregulated reward-seeking behavior in children and adolescents with ADHD [2,3]. Although not completely consistent [4,5], the majority of studies suggest that ADHD is associated with delayed aversion, an abnormal preference for immediate rather than delayed reinforcement, as well as a greater behavioral sensitivity to reinforcement contingencies, which is generally accompanied by lower psychophysiological reactivity (see [6] for review). On the neural level, subjects with ADHD were shown to have reduced striatal brain activation relative to controls when anticipating monetary rewards [7].

However, the majority of studies have mainly focused on the effects of tangible rewards such as money, tokens or food incentives, and specifically how tangible reinforcement is able to improve cognitive control performance in subjects with ADHD [6]. For instance, Konrad and colleagues [8] had found that inhibitory control in children with ADHD during a Stop Signal Task could be ameliorated through non-social incentives (i.e., tokens), improving task performance to the same level as normal controls.

Focusing on the effects of non-social or tangible rewards on cognitive control (e.g., response inhibition) neglects how emotionally loaded, social stimuli may affect cognitive control performance in patients with ADHD. Recently, we demonstrated that not only money, but also social incentives such as smiles and friendly faces act as effective reinforcers in a go/no-go task, boosting response inhibition accuracy in developing boys between 8 to 12 years of age [9]. Monetary incentives had a substantially stronger effect in improving inhibitory performance than social incentives, suggesting that social rewards do not have an equally strong reinforcing value compared to financial rewards in normal subjects. Geurts and colleagues [10] had recently investigated the effects of social motivation on cognitive control performance in children with ADHD by manipulating task instructions (subjects were told to compete with peers). In the social motivation condition, subjects with ADHD were able to increase their performance to the level of normal controls. No significant interaction between motivation and trial type was detected in this study, indicating that social motivation had a more general effect on task performance and was not specific to cognitive control accuracy. These data suggest that ADHD children may profit from monetary reward conditions, and social motivation can enhance cognitive performance in subjects with ADHD.

Thus, the aim of our study was to directly compare the effects of social (positive facial expressions) and non-social (monetary) rewards on cognitive control processes in subjects with ADHD. Testing how different reinforcers can effectively modulate cognitive control will improve future therapeutic intervention in affected patients.

We chose a task in which cognitive control abilities, such as response inhibition, were specifically modulated by different reward contingencies [9]. For this purpose, an incentive go/no-go task was applied, in which correct inhibitions were either socially or monetarily rewarded to a group of children and adolescents with ADHD and compared to healthy controls between 8 to 13 years of age. Based on previous findings [6,10], we hypothesized that social and monetary rewards could enhance cognitive control accuracy in children (i.e., reduce false alarms), but subjects with ADHD would have an improved response inhibition over healthy controls under conditions of non-social and social reinforcement. For both groups we expected to find that monetary incentives would improve response inhibition more than social incentives [9].

It has to be noted that the terms 'reward' and 'reinforcement' are used interchangeably throughout the paper (for a discussion concerning differences and overlaps of both concepts, see [3]). We formally define rewards as behavioral reinforcers if they appear contingent upon an action or response, and, thus, increase the probability of the specific response being executed in the future.

## Method

### Subjects

A total of 16 boys with ADHD and 16 healthy male controls participated in the study with both groups ranging in age from 8 to 13 years (M = 10.4, SD = 1.4). Only participants with an IQ = 85 (based on the WISC-III) were included in this study.

Children in the ADHD group met DSM-IV criteria for ADHD [11] and were recruited from the Outpatient Unit at the Department of Child and Adolescent Psychiatry. Healthy controls were recruited from local primary or grammar schools. All participants underwent an extensive psychiatric examination conducted by an experienced child psychiatrist using a German semi-structured parent interview (K-DIPS; [12]). In addition, parents evaluated their child's behavior using the Child Behavior Checklist (CBCL; [13]) and the German parental report on ADHD symptoms (FBB-HKS; [14]). The number of items from the FBB-HKS questionnaire equates to the number of DSM-IV items and also provides a severity score for each ADHD symptom. Psychiatric classification of individuals with ADHD was based on the diagnostic interview (K-DIPS), the developmental history of the child, playroom observations, pediatric examinations, and parent questionnaires (FBB-HKS, CBCL). Only children who fulfilled diagnostic criteria of DSM-IV in the K-DIPS were included in the ADHD group. CBCL ratings of the inattention/hyperactivity subscale were above a T score of 65 in all ADHD cases.

Seven participants with ADHD were coded as inattentive-only subtype, and nine children satisfied criteria for the combined subtype (two with comorbid oppositional defiant disorder/ODD; none fulfilled criteria of conduct disorder/CD). Exclusion criteria included any potentially confounding diagnoses such as obsessive-compulsive disorder, psychosis, mania, major depression, substance abuse, pervasive developmental disorders or developmental disorders. None of the ADHD participants used any medication other than stimulants, which were discontinued at least 48 hours prior to testing.

Informed consents were obtained from all participants and their parents. This study was approved by the Ethics Committee of the University Hospital, Aachen.

Demographic data and descriptive statistics for task performance of the two groups are summarized in Table 1. The groups did not differ with respect to age or IQ (all *p*s > 0.1). However, children and adolescents with ADHD were rated as having significantly higher inattention, externalizing and internalizing scores as assessed with the CBCL, and stronger ADHD symptom severity in the FBB-HKS (all *p*s = 0.01).

**Table 1:** Main group characteristics and overall performance on the incentive go/no-go task

	ADHD group (*n *= 16)	Control group (*n *= 16)	Group differences^a^
		
	M (SD)	M (SD)	
Age (years)	10.7 (1.6)	10.2 (1.3)	ns
IQ (WISC-III)	97.1 (14.2)	102.7 (9.5)	ns
CBCL (parent rating)			
Internalizing	60.4 (7.7)	52.3 (10.7)	*p *= .01
Externalizing	64.1 (4.8)	52.9 (6.6)	*p *< .001
Attention	71.1 (5.6)	54.4 (3.7)	*p *< .001
FBB-HKS (parent rating)	82.6 (10.4)	36.7 (18.9)	*p *< .001

FA baseline	29.0 (10.7)	30.0 (13.9)	ns
FA social reward	13.1 (7.3)	22.1 (10.9)	*p *= .011
FA monetary reward	10.6 (8.5)	14.8 (10.8)	ns
RT hits baseline	446.3 (84.3)	435.8 (46.1)	ns
RT hits social reward	438.8 (88.7)	414.6 (43.2)	ns
RT hits monetary reward	454.4 (80.4)	416.8 (58.4)	ns
RT false alarms baseline	355.2 (75.5)	407.5 (134.3)	ns
RT false alarms social reward	396.4 (121.1)	401.3 (126.0)	ns
RT false alarms monetary reward	397.9 (65.9)	388.7 (164.5)	ns

Note: ADHD = Attention-deficit/hyperactivity disorder; CBCL = Child Behavior Checklist (T scores are reported); FBB-HKS = German Parental Report on ADHD symptoms (percentiles); FA = false alarm rate (%); RT = Reaction time (in msec).

^a^Two-tailed t-tests.

### Experimental procedure

#### Incentive go/no-go task

In our incentive go/no-go task (see [9] for details), participants were instructed to respond with their dominant hand as quickly as possible for all go signals (letters "A" through "E"), but to inhibit a response for all no-go signals (letter "X"). The stimuli were pseudorandomly presented in the center of the computer screen for 500 msec with a fixed intertrial interval (ISI) of 1500 msec. Feedback – uninformative or informative (see below) – was given after no-go trials and was shown 1500 msec after the offset of the no-go signal for an additional 1500 msec.

The incentive go/no-go task consisted of two experimental blocks, each block with 150 trials (60% go signals and 40% no-go signals). In the first experimental block, all children underwent the same non-reward baseline condition where meaningless feedback (represented by uninformative mosaic pictures) was given for both successful and failed response inhibitions. In the second experimental go/no-go block, children from both groups were reinforced for successful inhibitions with social or monetary rewards presented block-wise. One reward block consisted of six rewards of the same type. Altogether 5 blocks of social (total number of trials = 30) and 5 blocks of non-social rewards (total number of trials = 30) were presented in a pseudorandom order. Happy and exuberant facial expressions served as positive social reinforcers, while neutral facial expressions were shown after false alarms (for validation of face stimuli, see [9]).

Correct inhibitions in non-social trials were positively reinforced with money, symbolized by different colored wallets each filled with a 50 Eurocent coin; empty wallets were shown after false alarms. Each child in the two groups won additional three Euros, irrespective of his performance, although he was informed that a better performance in the money condition would result in a higher amount of money paid after the experimental session.

Participants were reminded at the beginning of each experimental block to react quickly while maintaining a high level of accuracy.

We did not incorporate response cost manipulations in our go/no-go task (e.g., losing money for false alarms in the non-social condition), since we primarily focused on motivational effects of rewarding stimuli and not on punishment or punishment avoidance.

To ensure that all children understood the task instructions, experimental blocks were preceded by 20 practice trials with opportunities to repeat trials, if needed.

After each block, children were required to complete a subjective rating questionnaire to assess self-reported motivation, insight into performance, and aspects of task manipulations.

#### Subjective rating questionnaire

Following each experimental block, children were interviewed with a rating questionnaire. The questionnaire was developed to assess self-reports on subjective experiences associated with performing different experimental manipulations. A 5-point Likert-type scale (ranging from 0 to 4) was applied. The children were asked (1) how much they were motivated doing the task prior to the start of the experimental procedure, (2) how motivating and (3) how difficult they found the task, (4) how satisfied they were with their performance, and (5) how rewarding they found the different feedback stimuli.

### Statistical analysis

Dependent measures of the incentive go/no-go task – false alarm rates (FA rates), reaction times for hits (RT for hits), and reaction times for false alarms (RT for false alarms) – were analyzed using a multivariate ANOVA model with group as the between-subjects factor (two levels: healthy controls, ADHD), and reward type as a within-subjects repeated factor (three levels: non-reward, social reward, monetary reward), followed by univariate ANOVAs. As age and IQ did not differ significantly between the groups and were not correlated with the dependent measures, these variables were not included as covariates in analysis of performance data. The alpha level was set at 0.05. In addition, effect sizes were calculated using partial eta square (η^2^_p_). Since omission errors were very infrequent (below 3%), they were not included in the analysis.

To analyze the effects of performance feedback on subjective rating scores, the Wilcoxon signed-rank test for related samples was employed. Mann-Whitney U-tests were applied to assess differences between groups. Concerning subjective motivation ratings, we specifically analyzed the differential changes in the two groups from the baseline to the reinforcement conditions using the non-parametric Pair Differences-U-test for two independent samples [15].

All statistical analyses were performed using SPSS version 14.0 (SPSS Inc., Chicago, Illinois).

## Results

### Subjective ratings

Subjects in both the ADHD and control groups started the experimental procedure equally motivated according to self-ratings (Mdn_ADHD _= 2, Mdn_Controls _= 3; Mann-Whitney U = 89.5, ns).

After the non-reward baseline condition, self-ratings revealed that both groups did not differ in their ratings of task difficulty (Mdn_ADHD _= 2, Mdn_Controls _= 2; Mann-Whitney U = 97.5, ns) or in their satisfaction with task performance (Mdn_ADHD _= 3, Mdn_Controls _= 2.5; Mann-Whitney U = 121.5, ns). However, children with ADHD rated the baseline condition as less motivating than healthy controls (Mdn_ADHD _= 1, Mdn_Controls _= 3; Mann-Whitney U = 41.5, *p *= 0.001).

**Figure 1:**
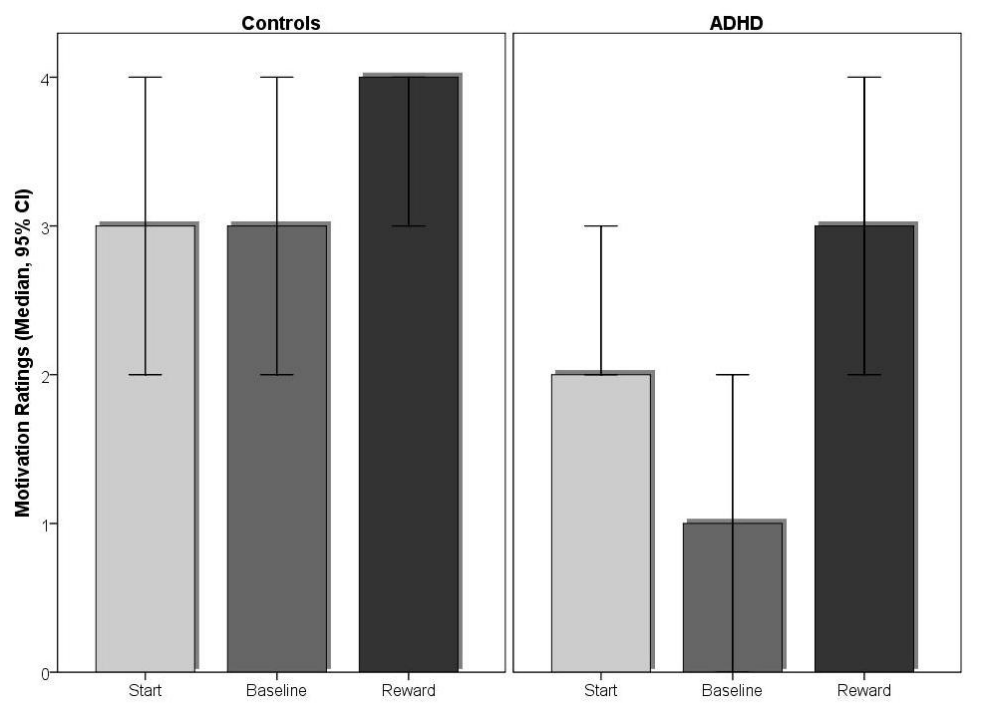
**Motivation ratings**. Motivation rating of the two experimental groups assessed at the start of the experimental procedure, after the non-reward baseline condition, and after the reward block. Motivation rating of the control group after the reward block indicates a possible ceiling effect. Median and 95% confidence interval (CI) are also depicted, as the distributions of the rating data were skewed.

After the reward block, children in both groups rated the task as more motivating and satisfying than the baseline condition, indicating that our experimental manipulation was successful (Self-rated motivation: Mdn_ADHD1 _= 1, Mdn_ADHD2 _= 3; Z = -3.1, *p *= 0.001; Mdn_Controls1 _= 3, Mdn_Controls2 _= 4; *Z *= -2.9, *p *= 0.002; Self-rated satisfaction: Mdn_ADHD1 _= 3, Mdn_ADHD2 _= 4; *Z *= -3.0, *p *= 0.001; Mdn_Controls1 _= 2.5, Mdn_Controls2 _= 4; *Z *= -2.6, *p *= 0.008). However, self-rated motivation was significantly lower in children with ADHD compared with healthy controls under reward conditions (Mann-Whitney U = 75.0, *p *= 0.037; see Figure 1).

Relating to possible differential changes of subjectively perceived motivation in the two groups from the baseline to the reward conditions, no significant differences were found (Pair Differences-U = 83.0, ns).

No differences between groups were detected for satisfaction with task performance (Mann-Whitney U = 101.5, ns) or subjectively perceived task difficulty (Mann-Whitney U = 88.0, ns).

When children rated the reward value of the feedback stimuli, both groups perceived the feedback after successful inhibitions as more rewarding in the reward block than in the baseline condition (Mdn_ADHD1 _= 2, Mdn_ADHD2 _= 3; *Z *= -3.3, *p *< 0.001; Mdn_Controls1 _= 2, Mdn_Controls2 _= 4; *Z *= -3.5, *p *< 0.001). We found no significant group differences either in the baseline or reward conditions. Note that we did not assess reward value ratings separately for social and monetary rewards.

### Task performance: False alarm rates and reaction times for hits and false alarms

Under the non-reward baseline condition, no significant differences in FA rates, RT for hits and RT for false alarms (F (3, 28) < 1, ns, η^2^_p _= 0.11) was observed between groups. Group characteristics and task performance for the two groups are summarized in Table 1.

The 2 × 3 (Group × Reward) repeated measures MANOVA with FA rates, RT for hits and RT for false alarms as dependent measures revealed a significant main effect for Reward, F (6, 25) = 33.46, *p *< 0.001, η^2^_p _= 0.89, and a significant Group × Reward interaction effect, F (6, 25) = 2.81, *p *= 0.031, η^2^_p _= 0.40, while the main effect for Group, F (3, 28) = 1.15, ns, η^2^_p _= 0.11, was found to be non-significant. This suggests that task performance in all children changed under conditions of reinforcement, but that rewards differentially affected performance in the two groups. Univariate ANOVAs showed that significant reward effects were only related to FA rates, F (2, 60) = 65.41, *p *< 0.001, η^2^_p _= 0.69, and RT for hits, F (2, 60) = 4.22, *p *= 0.019, η^2^_p _= 0.12, but not to RT for false alarms. The significant Group × Reward interaction effects were related to all three dependent measures (FA rates: *p *= 0.033, η^2^_p _= 0.11; RT for hits: *p *= 0.03, η^2^_p _= 0.11; RT for false alarms: *p *= 0.041, η^2^_p _= 0.10).

**Figure 2:**
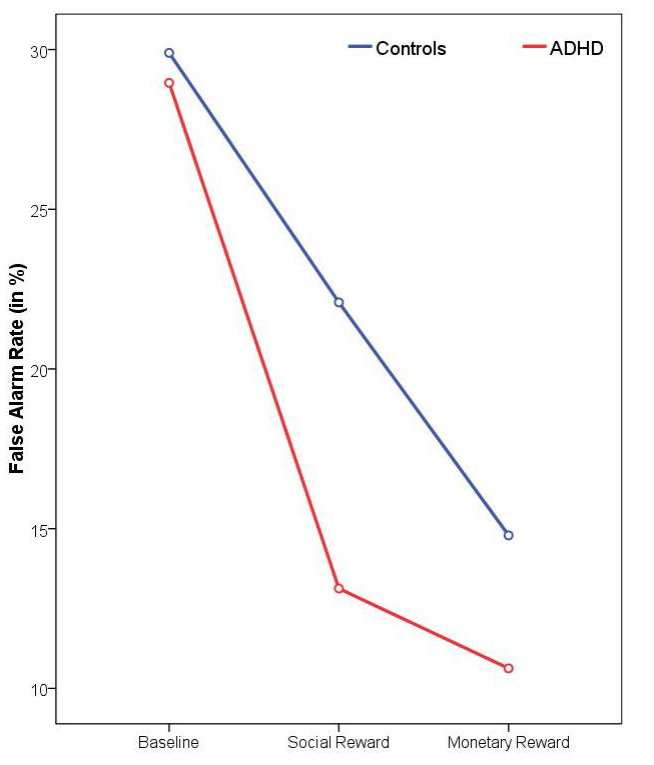
**False alarm rates**. Changes in false alarm rate (%) from the baseline to the two reward conditions for both groups.

As shown in Figure 2, the Group × Reward interaction for FA rates can be explained by a higher responsiveness of ADHD subjects to social (*p *= 0.019, η^2^_p _= 0.17) but not monetary rewards, suggesting that response inhibition accuracy can be improved more by social incentives in children with ADHD compared to healthy controls.

**Figure 3:**
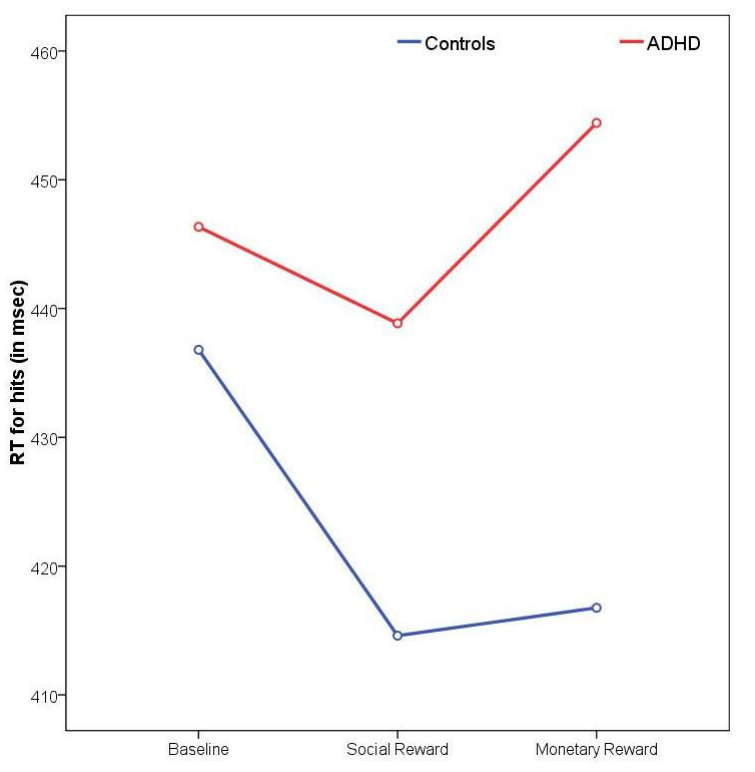
**Reaction times for hits**. Changes in reaction times for hits (in msec) from the baseline to the two reward conditions for both groups.

**Figure 4:**
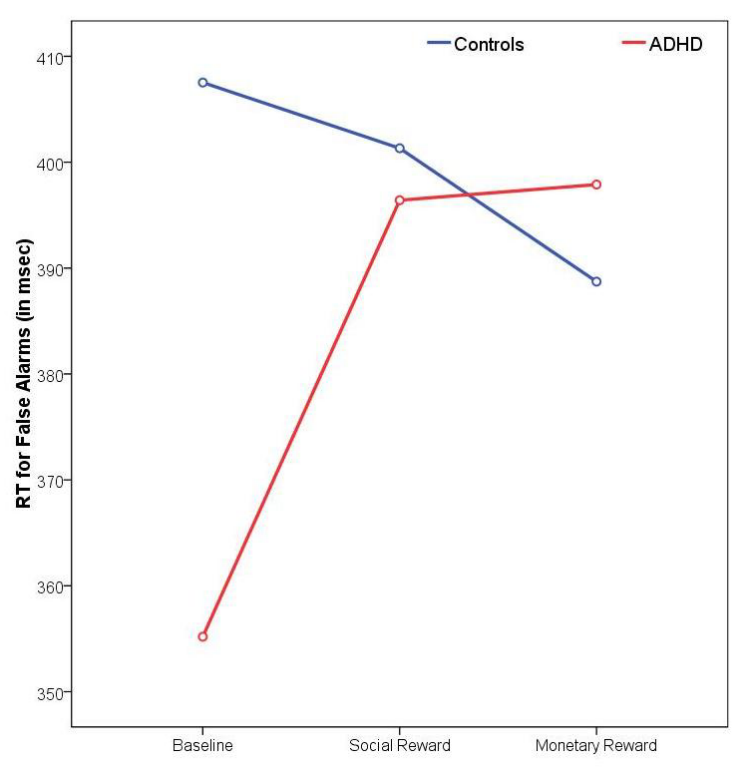
**Reaction times for false alarms**. Changes in reaction times for false alarms (in msec) from the baseline to the two reward conditions for both groups.

By contrast, the Group × Reward interaction effects for RT for hits and RT for false alarms were related to the monetary reward condition (RT for hits: *p *= 0.01, η^2^_p _= 0.19; RT for false alarms: *p *= 0.007, η^2^_p _= 0.22), but not to the social reward condition. Children with ADHD responded significantly slower (RT for hits) and less impulsively (RT for false alarms) under monetary reinforcement, whereas control subjects responded generally faster when money was at stake (see Figure 3 and 4).

### ADHD subtype analyses

Under the non-reward baseline condition, no significant differences in FA rates, RT for hits and RT for false alarms (F (3, 12) = 1.1, ns, η^2^_p _= 0.21) emerged between ADHD subtypes. Furthermore, no significant subtype by reward interaction effects with regard to differential benefit from social and non-social reward could be found.

### Possible changes in performance strategies

Possible speed-accuracy trade-off effects were inspected by calculating correlations between FA rates and RT for hits within each group for the three experimental conditions tested. All correlation coefficients were found to be non-significant (*p*s > 0.1), suggesting that children did not slow down reaction times for go signals to improve inhibition accuracy.

We also did not find significant associations between FA rates and RT for false alarms within each group for the three experimental conditions tested (*p*s > 0.3).

**Figure 5:**
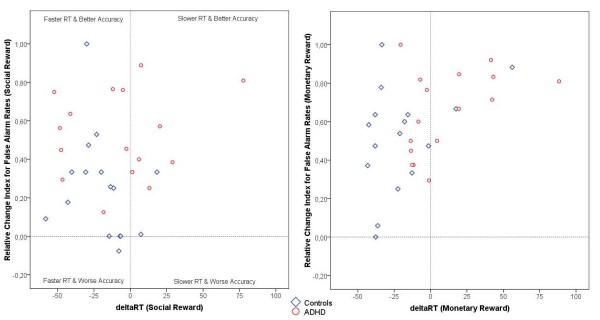
**Performance strategies**. Scatterplots (with reference lines at *x *= 0 and *y *= 0) show associations between ΔRT and relative change index for false alarm rates within each of the two groups and for the two reward conditions. Positive ΔRT values indicate RT slowing from baseline to the reward condition, and positive false alarm rates indicate improvement of inhibition performance from baseline to the reward condition. Social reward: control group, *r *= -0.26, *p *= 0.33; ADHD group, *r *= 0.14, *p *= 0.59. Money reward: control group, *r *= 0.37, *p *= 0.16; ADHD group, *r *= 0.44, *p *= 0.09.

With respect to changes in performance strategies between the baseline and both reward conditions, we also controlled for associations between ΔRT (RT differences between baseline and reward conditions) and the individual change index for false alarm rates (for details of these measures, see [9]) within each group and separately for the two incentive conditions. Again, all correlation coefficients were found to be non-significant (*p*s > 0.05), suggesting that differential improvements in inhibition accuracy between the baseline and incentive conditions cannot simply be explained by changes in performance strategies between the two groups. As illustrated in Figure 5, virtually all subjects in both groups (with the exception of one boy from the control group under social reinforcement) became faster and more accurate under the two reward conditions. None of the subjects showed increased RTs and reduced accuracy when rewards were at stake. However, there were a small group of control individuals and a substantial amount of ADHD subjects (on the right side of the reference line *x *= 0 and above *y *= 0), who obviously changed their response behaviors by slowing down reaction times to improve inhibition accuracy in the social as well as in the monetary reward condition (control group: *n *= 2 (12.5%); ADHD group: *n *= 7 (43.8%)).

## Discussion

This study is the first to compare the effects of tangible and non-tangible reinforcers on cognitive control accuracy in ADHD subjects. For this purpose, we employed an incentive go/no-go task to explore the extent to which social (affirmative facial expressions) and non-social (monetary) rewards differentially impact response inhibition in children and adolescents with ADHD compared to healthy controls. As expected, we found that both social and monetary contingencies improved cognitive control performance (i.e., reducing false alarms) with best task performance during the monetary reward condition in all participants, confirming earlier findings [9,16]. However, while the benefit from monetary reward was similar in both groups, individuals with ADHD showed a particularly higher profit from social reward compared to healthy controls, suggesting that cognitive control accuracy can be specifically improved by social reinforcement in ADHD patients. The results for the two included ADHD subgroups were the same as the results for the entire ADHD group. By contrast, self-rated motivation associated with task performance was significantly lower in subjects with ADHD. Additionally, ADHD subjects and healthy controls showed similar changes in their subjectively perceived motivation from baseline to the reward conditions, which is in line with previous reports [6]. Children and adolescents with ADHD had significantly slower reaction times under conditions of monetary reinforcement, while healthy controls generally reacted faster when money was at stake.

Our data partially replicate earlier findings that ADHD is associated with greater behavioral sensitivity to reinforcement contingencies [6,8,10,17]. The ADHD group showed a higher sensitivity to social but not to financial reinforcers when inhibition accuracy improvement is considered, which is in agreement with previous findings that children with externalizing disorders demonstrate greater enhancement of task performance under social reinforcement conditions than typically developing children [10,18,19]. Geurts and colleagues [10] reported that individuals with ADHD, who performed an interference control task, exhibited a significantly higher level of accuracy in a social reward situation (competing against fake peers) compared to a neutral condition, while normal controls showed no improvement. Likewise, but independent from reward processing, Krauel et al. [20] showed that social-emotional stimuli (relative to non-social stimuli) particularly had an augmenting effect on memory performance in ADHD patients, associated with increased parietal brain activation as revealed by functional MRI. Taken together, one may speculate that social-emotional stimuli, including social reinforcers, are specifically suited to enhance the arousal level in subjects with ADHD, which may help compensate for performance deficits on a variety of cognitive tasks [10,21].

Alternatively, but not mutually exclusively, it is also plausible that children with ADHD seek more social approval since they are frequently exposed to social disapproval by parents, siblings, and peers during interpersonal interactions and particularly in achievement situations [22]. A lack or 'deprivation' of positive social reinforcement in patients might result in stronger approach tendencies to receive social rewards [23]. Additionally, this could have produced greater improvements in cognitive control performance of individuals with ADHD, as found in this study. However, future research is needed to address this assumption. Moreover, it would be important to determine the extent to which social punishment, with respect to avoidance of social disapproval, is also able to enhance inhibitory control more in ADHD subjects than in typically developing control individuals.

Contrary to our prediction, we did not find a larger effect of monetary reinforcement on inhibition accuracy improvement in ADHD subjects compared to healthy controls. We could only detect that money differentially influenced reaction times in both groups, with slower RTs in ADHD children and faster RTs in healthy controls under financial reinforcement. The unexpected finding that money did not ameliorate response inhibition more in subjects with ADHD than in controls was similar to other studies that applied incentive go/no-go tasks [24,25]. Nevertheless, this is in contrast to data reported by Konrad and colleagues [8], who found that inhibitory control in children with ADHD performing a Stop Signal Task could be enhanced through non-social incentives (i.e., tokens), aligning stop-signal reaction times to that of healthy controls. However, a closer look at the task design reveals that, beside the non-social tokens, social praise (e.g., "Super!") was additionally used as reinforcement. This may explain the strong amelioration effect found in the ADHD group in Konrad's study. Taken together, it appears that the type of applied incentive, particularly social rewards, as well as the amount of reinforcement (see also [17]) play an essential role in enhancing task performance in subjects with ADHD.

Furthermore, the discrepancy between lower self-rated motivation and higher sensitivity to social response contingencies in the ADHD group suggests poor insight into realistic task monitoring, supporting other findings of limited self-awareness abilities in this patient group [26].

In contrast to previous studies that proposed deficient inhibitory mechanisms associated with frontostriatal brain abnormalities in ADHD patients [27,28], we did not find any deficit in response inhibition in our ADHD group. Although insufficient motor inhibition is often reported for this disorder (for reviews, see [29,30]), numerous studies failed to support disinhibition theories of ADHD ([24,25,31-33]; for a critical discussion concerning the use of "response inhibition" as an explanation of ADHD symptoms, see [3]). However, results of the present small-sample study are restricted due to limited statistical power and neuropsychological heterogeneity within the ADHD group.

Additionally, we did not investigate the underlying neurobiological mechanisms of reward responsiveness in ADHD subjects. Recent imaging data [7,34] suggest that neural *hypo*responsiveness in the mesolimbic dopaminergic brain circuit (including the ventral striatum) may be responsible for behavioral *hyper*responsiveness to incentives, mirrored in impulsive seeking of immediate reinforcers as a compensation strategy. Although these imaging studies exclusively employed monetary incentives, the findings may also hold for social incentives. Future studies would benefit from brain imaging techniques (fMRI, ERP) to increase our understanding of the neural basis of hypersensitivity to social incentives and its relation to behavioral symptoms in subjects with ADHD.

Higher responsivity to social reinforcers, such as positive facial expressions, in ADHD children is particularly noteworthy since various studies have reported emotion recognition and/or theory-of-mind deficits in affected children [35,36]. However, the findings of an emotion-processing deficit are inconsistent [37,38] and seem to be at least partly related to attentional dysfunction in subjects with ADHD [39,40]. Moreover, most researchers have noted problems with negative emotions such as anger, fear or disgust [41,42], but not with happiness, the positive social reinforcer used in our study. Our data do not point to an emotional deficit, as we found the strongest modulation of task performance by emotional expression in the ADHD group.

## Limitations

Our study has some limitations that should to be considered. First of all, as mentioned by Johansen and colleagues [43], given the heterogeneity within the ADHD population, it is arguable that dysregulated reward-seeking behavior alone can account for all cases of ADHD. Nevertheless, reinforcement theories are able to explain most of the ADHD symptoms [44]. ADHD possibly represents the final outcome of diverse and discrete neurodevelopmental pathways with an 'extreme reward approach pathway' leading to impulsive and overactive behavior [2].

One main shortcoming of this study previously mentioned relates to our sample group and size, which consisted solely of 32 young boys. Our conclusions are thus limited and in need of replication with a larger, more diverse sample group.

Although no clear subtype differences concerning reward responsiveness were previously reported for ADHD (see [6]), and were also not found in this study, future research should consider that possibly the ADHD hyperactive/impulsive subtype might differentially profit from social reinforcement.

Moreover, we did not include a clinical comparison group, which limits the specificity of the present findings. Future studies could benefit from the use of both non-social and social incentives to explore how different types of response contingencies work in different psychiatric disorders. For instance, application of the incentive go/no-go task on children with autism spectrum disorder will help to obtain deeper insights into the nature of a hypothesized hyporesponsivity to social reinforcement in this patient group [16].

It should be also noted that in our task design uninformative mosaic pictures were given for both successful and failed response inhibitions in the baseline condition, whereas in both reward conditions informative response contingencies were provided. Thus, it could be possible that the mere fact of having a feedback about task performance in the reward conditions compared to the non-reward baseline might have contributed to the higher reward value ratings of both reinforcement types in the subjective self-reports. However, most importantly, it is unlikely that this possible bias accounts for the impact of reward on task performance: Both social and non-social reinforcers served as performance feedback, but influenced response behavior differently.

## Conclusion

In sum, this is the first study that investigated the extent to which social (positive facial expressions) and non-social (monetary) rewards differentially impact cognitive control in subjects with ADHD. Concerning response inhibition improvement, external social reinforcement was more beneficial for the ADHD group than for controls. We extend earlier findings and interpret this result as a hyperresponsiveness to social rewards in ADHD subjects [10,18]. Such a motivational abnormality may severely impact interpersonal interactions between patients and their social environment. According to social exchange theory, seeking social rewards prompts people to establish mutual social interactions [45]. Hyperresponsiveness to social reinforcement likely disrupts balanced social interactions through impulsive acts [2] and has serious consequences on interpersonal relationships [46].

## Competing interests

The authors declare that they have no competing interests.

## Authors' contributions

GK designed and coordinated the study, collected and analyzed data and drafted the manuscript. BHD participated in the supervision of the project and commented on the written drafts of the manuscript including interpretation of the results. KK supervised the project, participated in the statistical analysis, corrected the drafted manuscript and contributed to the interpretation of the results. All authors read and approved the final manuscript.
